# Supplementary material for: A Nomogram for Predicting Non-Response to Surgery One Year after Elective Total Hip Replacement
Source: J Clin Med. 2022 Mar 16;11(6):1649. doi: 10.3390/jcm11061649 (PMC8955143; doi:10.3390/jcm11061649)
Supplement: Supplementary file 1 [file jcm-11-01649-s001.zip › Supplementary Figure S1.pdf]

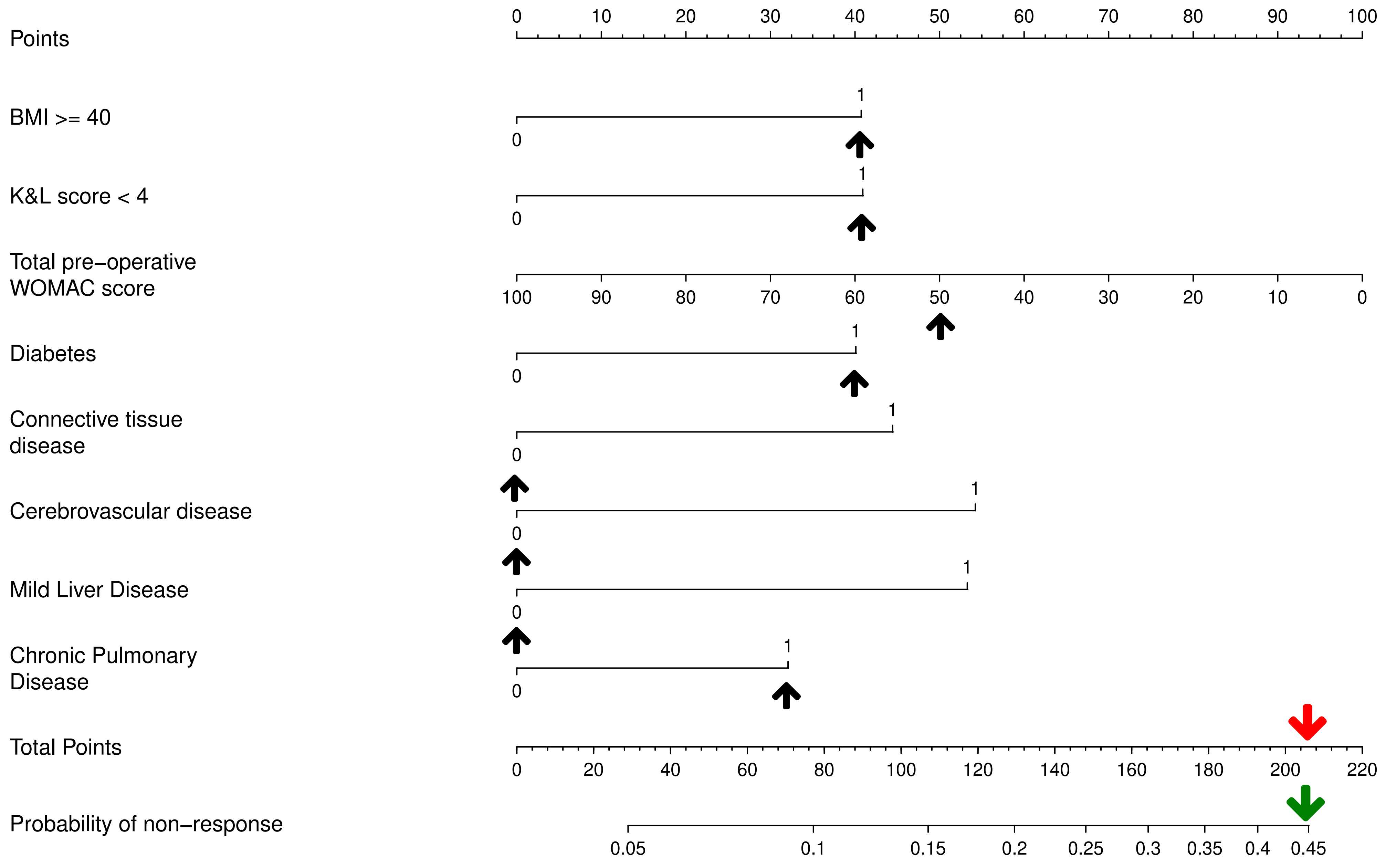

Points
0 10 20 30 40 50 60 70 80 90 100
BMI >= 40
0 1
K&L score < 4
0 1
Total pre−operative WOMAC score
100 90 80 70 60 50 40 30 20 10 0
Diabetes
0 1
Connective tissue disease
0 1
Cerebrovascular disease
0 1
Mild Liver Disease
0 1
Chronic Pulmonary Disease
0 1
Total Points
0 20 40 60 80 100 120 140 160 180 200 220
Probability of non−response
0.05 0.1 0.15 0.2 0.25 0.3 0.35 0.4 0.45

FIGURE LEGEND

Supplementary Figure S1: – Worked example of how to use nomogram.

First match each explanatory variable with the corresponding number of points on the top "Points" scale (black arrows). For example, a patient with a BMI≥40 kg/m^2 matches to 41 points, a K-L Grade of 3 corresponds to 41 points, a baseline WOMAC score of 50 matches to 50 points, comorbid diabetes corresponds to 40 points and chronic pulmonary disease matches to 33 points. This sums to a cumulative total of 205 points (red arrow). Drawing a line down from the "Total Points" scale to the corresponding "Probability of non-response" scale reveals that 205 total points corresponds to a non-response probability for this particular patient of 45% (green arrow).
