# Supplementary material for: A Nomogram for Predicting Non-Response to Surgery One Year after Elective Total Hip Replacement
Source: J Clin Med. 2022 Mar 16;11(6):1649. doi: 10.3390/jcm11061649 (PMC8955143; doi:10.3390/jcm11061649)
Supplement: Supplementary file 1 [file jcm-11-01649-s001.zip › Supplementary tables.pdf]

Supplementary Table S1: Clinical characteristics

Variable	Overall (N=2,177)	Responder (N=1,983)	Non-Responder (N=194)	p-value
Contralateral THR, [N (%)]				
Yes	596 (27.4)	531 (89.1)	65 (10.9)	.045
No	1,581 (72.6)	1,452 (91.8)	129 (8.2)	
Approach [N (%)]				
Anterior	486 (22.3)	457 (94.0)	61 (6.0)	.003
Hardinge	713 (32.8)	628 (88.1)	29 (11.9)	
Posterior	900 (41.3)	828 (92.0)	72 (8.0)	
Superpath	78 (3.6)	70 (89.7)	8 (10.3)	
Cementation [N (%)]				
Uncemented	706 (32.4)	655 (92.8)	51 (7.2)	.122
Hybrid	1,355 (62.2)	1,221 (90.1)	134 (9.9)	
Totally Cemented	114 (5.2)	105 (92.1)	9 (7.9)	
Femoral Head Size, [N (%)]				
22	7 (0.3)	6 (85.7)	1 (14.3)	.682
28	181 (8.3)	168 (92.8)	13 (7.2)	
32	1,406 (64.6)	1,283 (91.3)	123 (8.7)	
36	583 (26.8)	526 (90.2)	57 (9.8)	
Length of Stay [mean (SD)]	4.5 (2.6)	4.5 (2.7)	4.7 (2.1)	.191
Discharge Destination				
Home	1,723 (79.1)	1,599 (92.8)	124 (7.2)	<.001
Rehabilitation	454 (20.9)	384 (84.6)	70 (15.4)	
Complication/Adverse event				
Clavien-Dindo Grade I	195 (9.0)	172 (88.2)	23 (11.8)	.139
Clavien-Dindo Grade II	159 (7.3)	139 (87.4)	20 (12.6)	.092
Clavien-Dindo Grade III	90 (4.1)	74 (82.2)	16 (17.8)	.003
Clavien-Dindo Grade IV	13 (0.6)	13 (100.0)	0 (0.0)	.258
Unplanned Readmission				
Yes	90 (4.1)	76 (84.4)	14 (15.6)	.207
No	2,087 (95.9)	1,907 (91.4)	180 (9.6)	

Supplementary Table S2: Charlson Comorbidities

Variable	Overall (N=2,177)	Responder (N=1,983)	Non-Responder (N=194)	p-value
Cerebrovascular disease	91 (4.2)	75 (82.4)	16 (17.6)	.003
Chronic pulmonary disease	159 (7.3)	137 (86.4)	22 (13.8)	.024
Congestive heart failure	51 (2.3)	47 (92.2)	4 (7.8)	.786
Connective tissue disease	126 (5.8)	108 (85.7)	18 (14.3)	.029
Dementia	14 (0.6)	13 (82.9)	1 (7.1)	.816
Diabetes	294 (13.5)	253 (86.1)	41 (13.9)	.001
Mild liver disease	37 (1.7)	30 (81.1)	7 (19.9)	.031
Myocardial infarct	131 (6.0)	117 (89.3)	14 (10.7)	.462
Peripheral vascular disease	25 (1.1)	23 (92.0)	2 (8.0)	.872
Peptic ulcer disease	52 (2.4)	44 (84.6)	8 (15.4)	.097
Any tumor	96 (4.4)	85 (88.5)	11 (11.5)	.370
Diabetes/end organ damage	31 (1.4)	27 (87.1)	4 (12.9)	.432
Hemiplegia	2 (0.1)	1 (50.0)	1 (50.0)	.041
Leukemia	2 (0.1)	2 (100.0)	0 (100.0	.658
Lymphoma	22 (1.0)	19 (86.4)	3 (13.6)	.434
Moderate or severe renal disease	86 (4.0)	75 (87.2)	11 (12.8)	.198
Moderate or severe liver disease	14 (0.6)	11 (78.6)	3 (21.4)	.099
Metastatic solid tumor	7 (0.3)	7 (100.0)	0 (0.0)	.407

Supplementary Table S3: Predictors of non-response to THR

				Nomogram Candidate 1		Nomogram Candidate 2	
**Predictor**	**Category**	**Unadjusted OR (95% CI)**	**p-value**	**Adjusted OR (95% CI)**	***p-value**	**Adjusted OR (95% CI)**	***p-value**
Age (years)		1.01 (1.00, 1.03)	.065				
Sex	Male	Reference					
	Female	1.03 (0.76, 1.40)	.829				
BMI (continuous)		1.02 (1.00, 1.05)	.050				
Obesity Class	<30kg/m2	Reference					
	≥30 - <35kg/m2	1.06 (0.74, 1.51)	.753				
	≥35 - <40kg/m^2	1.24 (0.79, 1.94)	.652				
	≥40kg/m^2	1.97 (1.21, 3.21)	.007				
BMI ≥40kg/m^2		1.87 (1.18, 2.98)	.008	1.83 (1.12, 2.97)	.015	1.88 (1.16, 3.05)	.011
ASA (continuous)		1.37 (1.09, 1.74)	.008				
ASA ≥3		1.45 (1.09, 1.96)	.014	NS			
K-L Grade	4	Reference		Reference			
	≤3	1.99 (1.47, 2.69)	<.001	1.93 (1.41, 2.63)	<.001	1.89 (1.39, 2.56)	<.001
Aetiology	Osteoarthritis	Reference					
	Rheumatoid arthritis	1.49 (0.77, 2.89)	.239				
	Avascular necrosis	1.43 (0.84, 2.34)	.189				
	Dysplasia	1.38 (1.01, 1.87	.415				
CCI (continuous)		1.25 (1.13, 1.38)	<.001				
CCI (categorical)	0	Reference		Reference			
	1	1.53 (1.06, 2.20)	.022	1.62 (1.12, 2.34)	.011		
	≥2	2.36 (1.64, 3.40)	<.001	2.54 (1.75, 3.69)	<.001		
Cerebrovascular disease		2.29 (1.29, 4.07)	.005			2.39 (1.33, 4.30)	.004
Chronic pulmonary disease		1.72 (1.05, 2.82)	.030			1.64 (1.00, 2.71)	.052
Congestive heart failure		1.11 (0.44, 2.79)	.818				

Connective tissue disease		1.90 (1.13, 3.21)	.016			1.99 (1.17, 3.39)	.011
Dementia		0.79 (0.10, 6.17)	.818				
Diabetes		1.83 (1.26, 2.66)	.002			1.86 (1.26, 2.75)	.002
Mild liver disease		2.44 (1.06, 5.61)	.036			2.28 (0.99, 5.27)	.053
Myocardial infarct		1.35 (0.75, 2.44)	.322				
Peripheral vascular disease		1.40 (0.41, 4.76)	.590				
Peptic ulcer disease		1.34 (0.56, 3.22)	.507				
Any tumour		0.74 (0.39, 1.42)	.372				
Diabetes/end organ damage		1.53 (0.56, 4.13)	.407				
Hemiplegia		Insufficient no's					
Leukaemia		Insufficient no's					
Lymphoma		1.62 (0.47, 5.64)	.445				
Moderate/severe renal disease		1.53 (0.81, 2.89)	.192				
Moderate/severe liver disease		2.82 (0.84, 9.46)	.094				
Metastatic solid tumour		Insufficient no's					
Pre-op VR12 PCS		0.99 (0.97, 1.01)	.366				
Pre-op VR12 MCS		0.99 (0.98, 1.00)	.206				
Pre-op WOMAC Pain		0.99 (0.98, 0.99)	.002				
Pre-op WOMAC Motion		0.99 (0.98, 0.99)	.023				
Pre-op WOMAC Function		0.99 (0.98, 0.99)	.001				
Pre-op WOMAC Global	per 10 units	0.87 (0.80, 0.94)	.001	0.86 (0.79, 0.94)	<.001	0.86 (0.79, 0.94)	<.001

*Hosmer & Lemeshow: p>0.05
